# A case study for assessing the utility of a decision tree based learning algorithm in mental health inpatient care quality management

**DOI:** 10.1192/j.eurpsy.2022.454

**Published:** 2022-09-01

**Authors:** R. Wernigg, M. Wernigg

**Affiliations:** 1 National Hospital Directorate-General , Department Of System Analysis And International Affairs, Directorate Of Quality Improvement, Budapest, Hungary; 2 National Institute of Mental Health, Neurosurgery and Neurology, A, Budapest, Hungary; 3 Budapest Technical University, Faculty Of It Engineering, Budapest, Hungary

**Keywords:** Quality management, healthcare indicators, Big Data, machine learning

## Abstract

**Introduction:**

There is limited knowledge about the potential role of machine learning (ML) in quality improvement of psychiatric care.

**Objectives:**

Our case study was to determine whether ML decision trees used on patient databases are suitable for focussing on specific patient population samples of mental healthcare quality audits. Populations were identified by patient and care provider variables, and the time of treatment. Outcomes were defined as hospital mortality, over-long hospitalization (over average +1SD or +2SD); and short hospitalization (under average -1SD; under 3 days).

**Methods:**

We conducted a Split Train Test in Python for our outcomes on national mental health inpatient turnover data (2010 through 2018 for training and 2019 for testing). A well-fitting decision tree had the area under the curve (AUC) of the receiver operating characteristic (ROC) >= 0.7, and specificity >= 0.9. Performing qualitative analyses of decision trees, we rejected the ones with little clinical relevance.

**Results:**

Decision trees fit well (AUC = 0.7 to 0.9; specificity = 0.7 to 1.0; sensitivity = 0 to 0.69). For hospital death cases, the decision tree had AUC = 0.86, no difference after controlling for the types of hospital units, and was clinically relevant. Models predicting over-long hospitalization fit well (AUC=0,9); however, controlling for care pathways, good fit and sensitivity both vanished. No valid models emerged for undertime discharges. The decision trees revealed unique combinations of variables.

**Conclusions:**

Our ML decision trees used on healthcare databases proved promising for focussing quality audit efforts. Narrative analysis for the clinical contexts of the decision trees is indispensable.

**Disclosure:**

No significant relationships.

